# Changes in the Cervical Microbiota of Women with Different High-Risk Human Papillomavirus Loads

**DOI:** 10.3390/v14122674

**Published:** 2022-11-29

**Authors:** Milena Camargo, Laura Vega, Marina Muñoz, Ricardo Sánchez, Manuel Elkin Patarroyo, Juan David Ramírez, Manuel Alfonso Patarroyo

**Affiliations:** 1 Molecular Biology and Immunology Department, Fundación Instituto de Inmunología de Colombia (FIDIC), Bogotá 111321, Colombia; 2 Centro de Investigaciones en Microbiología y Biotecnología–UR (CIMBIUR), Universidad del Rosario, Bogotá 112111, Colombia; 3 Faculty of Medicine, Universidad Nacional de Colombia, Bogotá 111321, Colombia; 4 Health Sciences Division, Main Campus, Universidad Santo Tomás, Bogotá 110231, Colombia; 5 Animal Science Faculty, Universidad de Ciencias Aplicadas y Ambientales (U.D.C.A), Bogotá 111166, Colombia; 6 Molecular Microbiology Laboratory, Department of Pathology, Molecular and Cell-Based Medicine, Icahn School of Medicine at Mount Sinai, New York, NY 10029, USA

**Keywords:** human papillomavirus, viral load, follow-up, next generation sequencing, cervical microbiota

## Abstract

The cervical microbiota is essential in female sexual health, and its altered states seem to have a central role in the dynamic of high-risk papillomavirus (hrHPV) infections. This study aimed to evaluate the variation in bacterial communities’ compositions according to hrHPV. We collected two samples per woman, with a difference of 12 ± 1 months between them, and performed a follow-up on 66 of these women. The viral load (VL) of the hrHPV was estimated by quantitative PCR (qPCR), then it was normalized (using the *HMBS* gene as reference) and transformed to the Log_10_ scale to facilitate the interpretation. The VL was categorized as Negative, without hrHPV copies; Low, less than 10^0^ hrHPV copies; Medium, between 10^0^ to 10^2^ hrHPV copies; and High, >10^2^ hrHPV copies. The microbiota composition was described through the Illumina Novaseq PE250 platform. The diversity analyses revealed changes regarding the hrHPV VL, where women with low VL (<10^0^ hrHPV copies) presented high diversity. The community state type (CST) IV was the most common. However, in women with high VL, a lower association with *Lactobacillus* depletion was found. *Lactobacillus gallinarum* and *L. iners* were the most abundant species in women with high VL, whereas women with low VL had a 6.06 greater probability of exhibiting *Lactobacillus* dominance. We identified conspicuous differences in the abundance of 78 bacterial genera between women with low and high VL, where 26 were depleted (e.g., *Gardnerella*) and 52 increased (e.g., *Mycoplasma*). A multilevel mixed-effects linear regression showed changes in the diversity due to the interaction between the measurement time and the VL, with a decrease in diversity in the second follow-up in women with low VL (Coeff. = 0.47), whereas the women with medium VL displayed an increase in diversity (Coeff. = 0.58). Here, we report for the first time that the cervical microbiota is influenced by the number of copies of hrHPV, where a decrease in the abundance of *Lactobacillus*, greater diversity, and enrichment of bacterial taxa is relevant in women with low VL.

## 1. Introduction

The relationship between persistence of high-risk human papillomavirus (hrHPV) infection and cervical cancer (CC) development has been shown [1]. However, many hrHPV infections become spontaneously resolved (6 to 18 months), while at least 25% remain persistent [1,2,3]. Host characteristics have been related to hrHPV persistence, including polymorphisms in human leukocyte antigen genes [4], sexual behavior (age at first intercourse and number of sexual partners) [5], alcohol consumption [6], nutritional habits, hormonal contraceptive use, among others [7].

Moreover, virus-specific factors favor its permanence and, consequently, CC development, including infections harboring multiple hrHPV types, which could act synergistically and favor its persistence in the host [8]. The hrHPV coexistence with other microorganisms in the same host might facilitate viral colonization [9,10]; the viral load (VL) also appears to play an essential role regarding hrHPV persistence [11,12].

The cervical tract is a relevant microecological region, and its environment can directly affect women’s sexual and reproductive health. In this sense, low diversity with high prevalence of certain microbial communities in the cervix are considered a sign of health and can limit the acquisition of pathogens, including hrHPV [13,14]. A healthy cervical microenvironment is dominated by *Lactobacillus* species (*Lactobacillus crispatus*, *L. gasseri*, *L. iners*, and *L. jensenii*), and the depletion in *Lactobacillus* communities along with an increase in anaerobic bacteria (e.g., *Gardnerella*, *Clostridiales*, and *Prevotella*) can lead to pathologies such as bacterial vaginosis (BV), hrHPV persistence, and progression of cervical abnormalities [13,14]. Four different community state types have been defined, depending on the predominant *Lactobacillus* species (type and abundance), and allow the categorization of the vaginal bacterial communities. CST I is dominated by *L. crispatus*, CST II has *L. gasseri* as the predominant species, CST III is dominated by *L. iners*, and CST V presents *L. jensenii* as the predominant species. On the other hand, CST IV is characterized by a low abundance of *Lactobacillus* species and increased abundance of anaerobic bacteria [15]. The CST classification is advantageous, as some studies have suggested associations between the CST and the host’s immune response, the acquisition of sexual transmission diseases, an unhealthy microenvironment, and pregnancy and neonatal outcomes [14,16,17].

Transversal studies have demonstrated the association between altered bacterial communities and the presence of HPV [17,18,19]; moreover, longitudinal studies have shown that dysbiosis is related to incident hrHPV infection and decreased viral shedding [14,20,21]. Despite advances in the understanding of microbial communities at the cervical level, and their association with hrHPV infections, more knowledge is required about the longitudinal dynamics and their relationship with the increase, or not, in the number of viral copies. Likewise, this would allow a more precise understanding of the impact of hrHPV persistence on microbial ecology and its contribution to the progression of cervical malignancy.

Therefore, we performed a longitudinal study that used qPCR to quantify and determine the persistence and clearance of infection by hrHPV [12], and to identify changes in microbial communities’ compositions in relation to HPV infection dynamics in a Colombian population. In this context, the study aimed at evaluating variations in the bacterial communities according to the follow-up (two follow-ups during a year of study), number of viral copies (low, medium, or high VL), and variation (decreased, equal, or increased) in the VL throughout the follow-up.

## 2. Materials and Methods

### 2.1. Study Population and Ethical Considerations

The original cohort study (which included 219 women) was carried out from 2007 to 2010, aimed at determining the natural history of HPV infection in women between 17 to 71 years old from the following healthcare centers: Hospital San Juan Bautista in Chaparral, Nuevo Hospital San Rafael in Girardot, and level II Engativá Hospital in Bogotá, Colombia. Inclusion/exclusion criteria for this HPV cohort was previously published [12,20]. All women included in the study signed an informed consent form authorizing sample use for both cohort study and future studies, including microbiota characterization (Figure S1). Women aged less than 18 years old and leading an active sexual life who had expressed their desire to participate in the study filled out the questionnaire and their consent were completed by the guardians responsible for them.

In the retrospective component of the study, we performed amplicon-based next generation sequencing to identify the microbial communities (Figure S1A). The inclusion criteria of the samples were (1) women that had two follow-ups (12 ± 1-month intervals), (2) HPV identification and quantification results, and (3) had a sample availability with the minimum amount of DNA as well as the required quality. Thus, we employed those samples with a higher concentration of 20 ng/μL to 260/280 ratio between 1.65 and 2.0, and with quality verification through agarose gel. Considering the mentioned inclusion criteria, 66 women were included, where each one contributed two samples at intervals of 12 months (±1-month).

All the women included in the study signed an informed consent form authorizing sample use for both the prospective and future studies. All procedures had been evaluated and approved by the level II Engativá Hospital’s Ethics Committee in Bogotá (CEHE-009) and were drawn up in line with the Declaration de Helsinki and Colombian Ministry of Health and Social Protection guidelines.

### 2.2. Molecular Detection and Quantification of hrHPV

The DNA was extracted employing the commercial kit QuickExtract^TM^, following the previously published protocol [12]. Subsequently, the detection and quantification of the hrHPV load was performed. As the first step, we performed, by conventional PCR (cPCR), HPV generic detection using primers targeting the L1 (GP5+/6+ and MY09/11) and E6/E7 regions (pU1M/2R), as has been published before [12,20]. In the next step, those samples positive for the generic HPV PCR were subjected to detection and quantified by the qPCR of six hrHPV types (hrHPV-16, -18, -31, -33, -45, -58); qPCR primers, probes (targeting the E1, E6, E7 regions), and conditions have been described previously [12,20]. Human hydroxymethylbilane synthase (HMBS) gene concentration was quantified; this was used as a host/housekeeping gene (considering the calculation of two housekeeping gene copies) for normalizing the number of viral copies to the human DNA quantity per sample. The calculation for this normalization has been published previously [12,20].

Once the quantification was normalized (considering the amount of HMBS gene copies), the viral load was transformed and presented in log_10_ to facilitate its interpretation. According to the percentage distribution of hrHPV, VL was categorized as follows: Negative, no hrHPV infection; Low VL, less than 10^0^ hrHPV copies; Medium VL, between 10^0^ to 10^2^ hrHPV copies; and High VL, ≥10^2^ hrHPV copies [12]. The total VL was obtained from the sum of the VL of each hrHPV detected in the study.

### 2.3. Illumina Sequencing and Bioinformatics Analyses

The hypervariable region V4 of the ribosomal gene 16S rRNA was sequenced for the 132 samples, using the primers 515-F (5′-GTGCCAGCMGCCGCGGTAA-3′) and 806-R (5′-GGACTACHVGGGTWTCTAAT-3′), specific for bacteria and archaea [22]. The amplification was carried out, followed by the construction of DNA libraries of microbial amplicons and adapter addition. Subsequently, these libraries were submitted to paired-end sequencing in the Illumina Novaseq PE250 platform to generate 250 bp raw reads, with a minimum expected depth of 100 thousand reads per sample. The demultiplexed reads were obtained, and the barcodes and primers were removed by implementing QIIME2 software (version 2019.7) [23,24].

The taxonomic assignation process was performed using the R package DADA2 following the recommended pipeline (https://benjjneb.github.io/dada2/tutorial.html, accessed on 15 November 2022). Briefly, the reads were filtered considering a Phred score equal to or greater than 30, and a merge of forward and reverse reads was performed. The central sample inference algorithm was used to infer the amplicon sequence variant (ASV) table, from which the chimeras of the sequences were removed. Finally, the first taxonomic assignation was executed using the SILVA v132.16s database. Next, we performed a double-check on the workflow and taxonomy assignments with DADA2 and SILVA version 138, considering a bootstrap of 50, according to the functions established in the DADA2 package [25,26].

Additionally, in the case of having a high percentage (~30%) of unclassified reads at the genus rank (“Unclassified”), we used BLASTn to perform an additional identification procedure. Hence, a reference database was generated with sequences corresponding to the ribosomal gene 16S rRNA (bacteria and archaea), including, specifically, the sequences of the curated database RefSeq. Simultaneously, we generated a multifasta file with the sequences cataloged as “Unclassified” to compare them against the constructed reference database, considering an e-value smaller than 10 and a percentage of identity above 95%. The table that resulted from this process was added to the relative abundance table obtained by the DADA2 pipeline to include all the information in subsequent analyses.

### 2.4. Descriptive and Statistical Analyses of the Bacterial Communities

Qualitative variables (origin, average monthly income, marital status, number of sexual partners, pregnancies, contraceptive method used, abortions, and colposcopy result) were expressed as frequencies and percentages. A measure of central tendency (median) was used for the quantitative variables (age and age at first intercourse), along with their measures of dispersion (interquartile ranges—RIC).

The data for bacterial community distribution and *Lactobacillus* are displayed according to the follow-up (baseline and follow-up at 12 months). For all the analyses, we considered two variables, where each one has different groups: viral load (categorized as low, medium, and high) and the viral load outcome through the follow-up (decreased, equal, increased). The viral load outcome during follow-up was counted despite the initial level of VL (Figure S1B).

Therefore, we performed alpha diversity analyses for the ASV identified in each of the groups contained within the three variables. For each group, the abundance-based coverage estimator (ACE) was calculated to estimate the bacterial richness, and the Shannon and Simpson indexes were calculated to estimate the bacterial diversity [27,28]. The statistical differences of richness and diversity between the groups contained within the follow-up variable were evaluated with a Mann–Whitney test (*p* < 0.05). In contrast, the statistical differences of richness and diversity between the groups contained within the viral load and viral load outcome variables were evaluated with a Kruskal–Wallis test and the post hoc Dunn test with a Benjamini-Hochberg correction (FDR). A multilevel mixed-effects linear regression was performed to analyze the effect of the measurement time and the viral load on the bacterial diversity. These methods make it possible to assess this effect at three different levels (first and second follow-up, viral load, and the interaction between these two variables).

The differences in the relative abundance of *Lactobacillus* between the groups (follow-up, viral load, and viral load outcome) were analyzed with the Mann–Whitney test or the Kruskal–Wallis test, depending on the number of groups to compare. For each sample, we determined the community state type (CST), considering the relative abundance of the *Lactobacillus* species. Thus, this was calculated according to previous reports, where the relative abundance of *Lactobacillus* should be higher than >60%, and aerobic and anaerobic bacteria display an abundance ranging from 14 to 40% [13,14]. Afterwards, conditional logistic regression was used for assessing the association between CST, *Lactobacillus* depletion, and the analyzed groups. Crude odds ratio (OR) and adjusted OR with their 95% confidence intervals (CI) were estimated, including as covariates (age and colposcopy result) in the model. STATA14 software was used for all two-tailed statistical tests, values of *p* < 0.05 were considered statistically significant.

For the beta diversity analyses, we performed a principal coordinates analysis with Bray–Curtis calculated distances. Permutational analysis of variance using distance matrices (adonis) was used to evaluate statistical differences of the sample clustering depending on the study group. Alpha and beta diversity analyses were carried out using the R phyloseq package [29].

### 2.5. Analyses of Differentially Present Genera and Correlation Network Construction

The DESeq2 package was implemented to assess significant differences in the abundance of the bacteria and archaea of each group contained within the three variables [30]. For this, a Wald test was applied to the dataset, and the differences in the abundance of the genera were detected by performing pairwise comparisons of the groups constituting the variables of interest. The differences were considered significant if the *p*-value was <0.01 (adjusted by Benjamini–Hochberg correction).

Finally, we constructed correlation networks with the 50 most abundant genera, considering the viral load and viral load outcome. First, a non-parametric Spearman correlation test was ran, considering a Benjamini–Hochberg correction and a correlation coefficient above 0.75 and below −0.75. Later, the networks were constructed using the R packages igraph, ggraph, and RCy3, and finally represented in Cytoscape v3.9.1. Descriptive analyses, statistical analyses, and generation of the corresponding figures were all performed by implementing R v4.1.0, along with the packages Vegan, corr, FSA, psych, igraph, ggraph, RCy3, tidyverse, reshape2, ggplot2, phyloseq, ranacapa, DESeq2, and ampvis2.

## 3. Results

### 3.1. Population Characteristics

A total of 66 women met the inclusion criteria, and each woman provided at least two samples, resulting in a total of 132 samples analyzed; the median follow-up interval was 377 days (IQR: 33). The women’s mean age at enrollment was 42.5 (IQR, 20) years old, and the mean age upon beginning sexual life was 18 years old (IQR, 5 years). Significant differences were observed in the distribution of contraceptive methods, the category of no contraceptive use being the most frequent among women with a medium initial VL. In contrast, the category of barrier methods and surgery were the most frequent among women with low and high initial VL (*p* = 0.046). In the same way, we observed a significant distribution in the results of the colposcopy, where the women with a low initial VL leaned towards a higher result of colposcopy abnormality at the beginning of the study (*p* = 0.047). Table S1 lists other categories and their descriptive statistics.

Regarding the VL (shown as log_10_-transformed), the median of the group with a low initial VL was −1.36 (1.10 IQR), for a medium initial VL was 1.32 (1.51 IQR), and for a high initial VL was 4.90 (3.87 IQR) (Figure S2). In the second follow-up, we found that the women with a low VL had a 2.85-fold greater probability of increasing their VL throughout the follow-up (McNemar’s chi-square = 0.0192) (Figure S3).

### 3.2. Cervical Microbiota Composition

We obtained an average of 381,403 reads per sample, with a minimum of 185,911 and a maximum of 618,286 reads. The statistics generated by the multiqc report showed that all the reads were of good quality on average (Phred score > 30), considering that the rarefaction analysis indicated a minimum of 200,415 reads to capture the diversity in most of the samples (Figure S4). The data for this study have been deposited in the European Nucleotide Archive (ENA) at EMBL-EBI under project accession PRJEB55513.

Through 16S rRNA (V4 region) amplicon sequencing, a total of 99,304 ASV belonging to the bacteria domain were identified and assigned to 62 phyla and 1908 bacterial genera (Tables S2–S4). On the other hand, from the total number of reads, only 1341 (1.33%) ASVs were classified as archaea (Table S5). Within the archaea, the most abundant genera were *Candidatus nanosalinarum* with 1178 assigned reads, *Methanobrevibacter* (22 reads), *Candidatus nitrosotalea* (21 reads), *Methanosaeta* (15 reads), and *Candidatus nitrososphaera* (14 reads) (Table S6). We performed a close-up analysis of the abundance of these archaea considering the viral load (Figure S6), where the individuals categorized with a high VL had an absence of archaea compared to those with a low or medium VL. Conversely, the individuals with a low VL displayed a variety of archaea genera compared to those with high or medium VL, in which a predominance of one genus was observed. Finally, we observed that the individuals in which the VL persisted or decreased had an absence of archaea compared to those with an increased VL (Figure S5).

### 3.3. Compositional Differences between Groups

**Figure 1:**
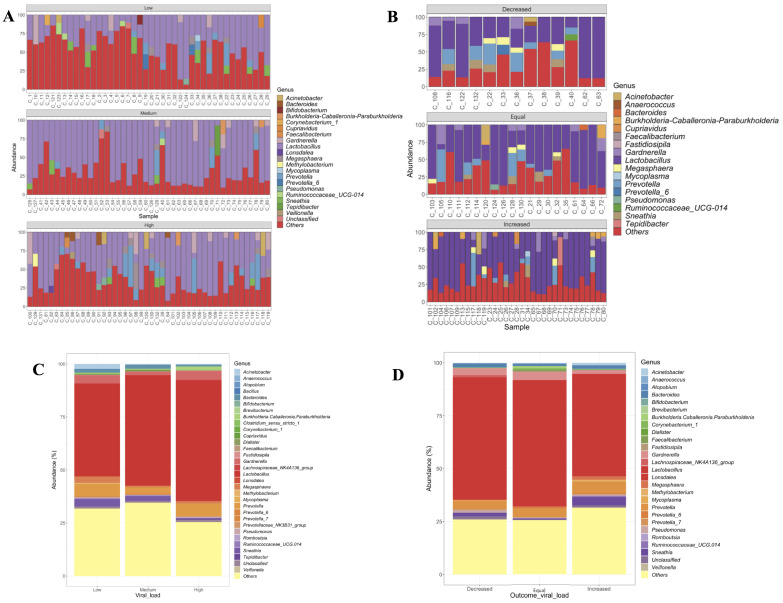
Microbial composition of cervical samples. (**A**) Bar plots showing the main bacterial genera by viral load, considering all the follow-up time points (first and second), and categorizing women according to their VL independent of their follow-up point. (**B**) Bar plots showing the major bacterial genera by viral load outcome. For this figure, the viral load outcome at 12 months is considered. (**C**) Distribution of each bacterial genera by viral load. (**D**) Distribution of each bacterial genera by viral load outcome.

The bacterial community composition varied between the follow-ups, exhibiting an increase in *Lactobacillus*, *Gardnerella*, and *Megasphaera* at the follow-up point of 12 months (Figure S5A,B). The description of the bacterial composition according to the VL showed that women with low VL were characterized by a decrease in *Lactobacillus* and an increase in *Megasphaera* and *Acinetobacter*, whereas women with a high VL had a higher relative abundance of *Lactobacillus*, *Gardnerella*, and *Burkholderia* (Figure 1A,C). Finally, regarding the behavior of the VL throughout the follow-up, those women that presented an increase in the VL (an increase independent of the initial level of VL) had a higher abundance of *Acinetobacter*, *Megasphaera*, *Prevotella*, and *Sneathia* (Figure 1B,D).

On the other hand, the relative abundances of the 20 main bacterial genera were compared regarding events evaluated; the descriptive results showed an increase in the relative abundance of *Lactobacillus* and *Gardnerella* in the group of women with a high VL compared to those with low VL (Figure S6A). Finally, women that exhibited a decreased VL throughout the follow-up had a low abundance of *Lactobacillus* and a high abundance of *Gardnerella* (Figure S6B).

### 3.4. Alpha and Beta Diversity Analyses

**Figure 2:**
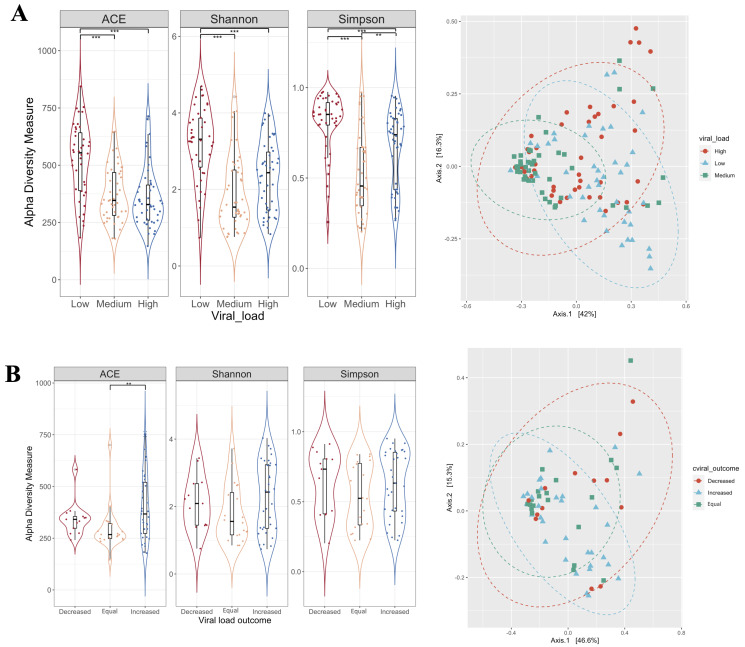
Alpha and beta diversity between groups. (**A**) Boxplot of diversity indexes and NMDS plot regarding viral load. (**B**) Boxplot of diversity indexes and NMDS plot regarding viral load outcome. Significant differences between the study groups were evaluated using a Kruskal–Wallis test ** *p* = 0.001–0.01, and *** *p* < 0.001.

To estimate alpha diversity for the ASV, we calculated the abundance-based coverage estimator (ACE) for the richness estimation, and Shannon–Weaver and Simpson indexes were calculated for the diversity estimation of the bacterial communities (Figure 2). The diversity analysis revealed a higher bacterial diversity (richness and abundance) in women with low VL (less than 10^0^ HPV copies) compared to women with high VL (Low vs. High. ACE, *p* = 0.0000056; Shannon, *p* = 0.00049; Simpson, *p* = 0.0021), and to women with medium VL (Low vs. Medium. ACE, *p* = 0.000062; Shannon, *p* = 0.00000018; Simpson, *p* = 0.000000047) (Figure 2A). Regarding the VL outcome, only significant differences were found in the richness, where women with an increase in the VL throughout the time had a higher richness compared to women with an equal VL throughout the study time (one-year follow-up) (*p* adjusted = 0.018, (Dunn test)) (Figure 2B).

The principal coordinates analysis (PCoA), based on Bray–Curtis dissimilarities, did not show a clear clustering of the analyzed groups (Figure 2, right hand). However, the PERMANOVA analysis using distance matrices (adonis) showed statistically significant differences in the distribution of the centroids according to the viral load (r^2^ = 0.11, *p* = 0.001) (Figure 2A). In the case of the VL outcome, we did not observe significant differences in the distribution of the centroids (r^2^ = 0.045, *p* = 0.15) (Figure 2B).

In addition, the effect of the variables (follow-up, viral load, and interaction between follow-up and viral load) on the bacterial diversity was evaluated (Table S7). Regarding the follow-up, the results showed a significant decrease in the diversity with respect to time (Coeff. = −0.41 units; *p* = 0.000) (Table S7). In contrast, the diversity reduced by 0.34 units, on average, in the group with medium VLs (*p* = 0.000) and 0.17 units in the group with high VLs, compared to the low VL group (*p* = 0.004) (Table S7). Subsequently, the evaluation of the interactions between the variables showed that the changes in the diversity through time vary according to the viral load. The highest decrease in the diversity was observed in the second follow-up in women with low VL (Coeff. = 0.47), compared to the women with low VL in the first follow-up (Coeff. = 0.89). Conversely, an increase in the diversity was observed in women with medium VL in the second follow-up (Coeff. = 0.58) compared to those in the first follow-up (Coeff. = 0.54) (Table S7).

### 3.5. Identification of Markers and Correlations between Bacterial Communities among the Groups Evaluated

**Figure 3:**
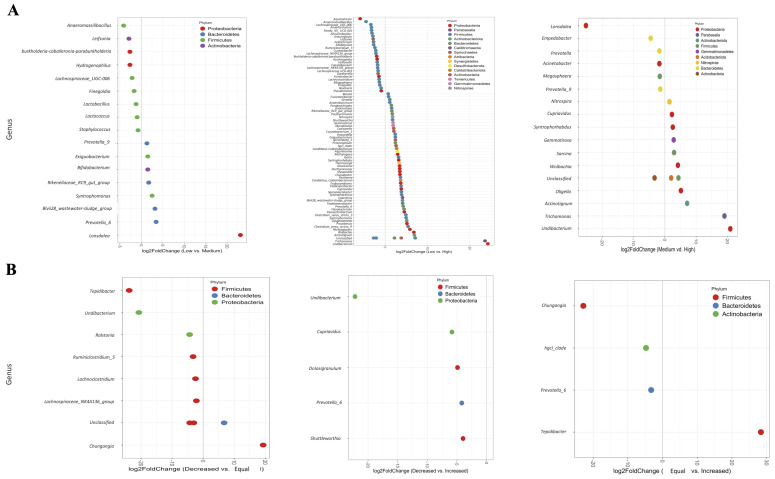
DESeq2 analysis for the identification of differentially abundant genera between groups according to viral load and viral load outcome. (**A**) Differentially abundant genera regarding viral load. Left and middle graphs consider the women with a low viral load as the point of comparison. The genera on the left side of the line are those with a decreased abundance in the women with low VL compared to those with medium or high VL. Conversely, the genera at the right side of the line are those with an increased abundance in the women with low VL. The right graph considers the women with a medium viral load as the point of comparison, where the genera on the left side of the line are those with a decreased abundance in the women with medium VL compared to those with low or high VL. Conversely, the genera at the right side of the line are those with an increased abundance in the women with medium VL. (**B**) Differentially abundant genera regarding viral load outcome. Left and middle graphs consider the women with a decreased viral load as the point of comparison, where the genera on the left side of the line are those with a decreased abundance in the women with a decreased VL, compared to those with an equal or increased VL. Alternatively, the genera at the right side of the line are those with an increased abundance in the women with a decreased VL. The right graph considers the women with equal VL as the point of comparison; thus, the genera on the left side of the line are those with a decreased abundance in the women with equal VL compared to those with an increased VL. Meanwhile, the genera at the right side of the line are those with an increased abundance in the women with equal VL.

The identification of differentially present genera in the two variables (VL and VL outcome) (Figure 3 and Figure S7) was developed by implementing DESeq [31]. In women with low VL compared to medium and high VL, we found a diminished abundance of the following genera: *Anaeromassilibacillus*, *Leifsonia*, *Lachnospiraceae* and *Burkholderia-Caballeronia-Paraburkholderia*. Meanwhile, *Prevotella*, *Exiguobacterium*, *Rikenellaceae*, *Blvii28*, and *Syntrophomonas* were significantly increased (Figure 3A, left graph). Interestingly, 78 genera were differentially abundant when comparing low VL with high VL (Figure 3A, middle graph); 26 genera were underrepresented in the low VL women compared to the high VL women. For instance, *Anaerocolumna*, *Ruminiclostridium*, *Faecalibacterum*, *Gardnerella*, *Megasphaera*, and *Pseudomonas* were found with decreased abundances. Meanwhile, we found 52 genera with an increase in their abundance in the women with low VL compared to those with high VL, for instance: *Nitrospora*, *Mycoplasma*, *Spirochaeta*, *Wolbachia*, *Trichomonas*, and *Unidibacterium* (Figure 3A, middle graph).

Next, we evaluated the differentially abundant genera according to the VL outcome throughout the time of the study (Figure 3B). The results show that *Tepidibacter* was diminished in women who decreased their VL compared to those with an equal VL (Figure 3A, left graph), while the abundance of this genus was higher in women with equal VL compared to women who increased their VL (Figure 3B, right graph). Finally, *Undibacterium* was the genus with the lowest abundance in women who decreased their VL through time compared to women with equal or increased VL (Figure 3B left and middle graph).

**Figure 4:**
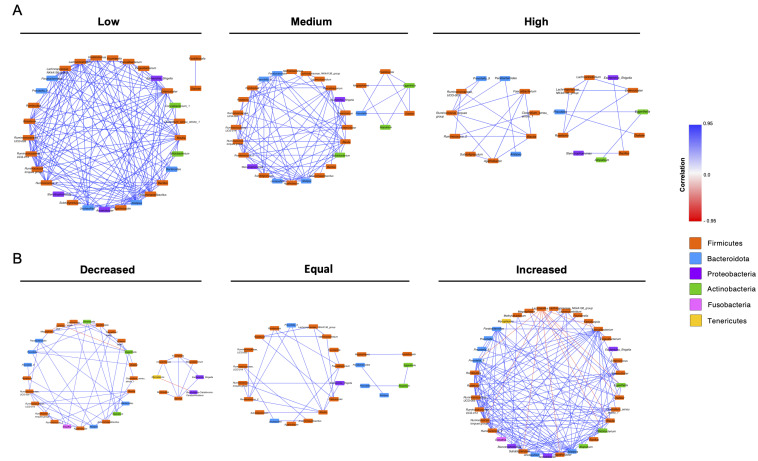
Correlation networks of the 50 most abundant genera of the study population. (**A**) Network visualization of pairwise correlations between genera according to viral load. (**B**) Network visualization of pairwise correlations between genera according to viral load outcome. Spearman correlation test was used to estimate correlation coefficients. The nodes represent the bacterial genera, and their color represents the phylum to which they belong. Positive correlations are illustrated as blue edges and negative correlations as red edges. The networks only represent significant correlations (*p* < 0.05, after FDR correction) with ρ above 0.75 and lower than −0.75, and that were computed with the Spearman correlation test.

To evaluate the possible interactions between bacterial communities in the groups constituting the two variables, we constructed correlation networks of the 50 most abundant genera (Figure 4). The networks that were constructed considering the VL displayed only direct correlations between the genera, where most of the genera belonged to the Firmicutes phylum. The network corresponding to the individuals with a high VL was the least densely connected compared to low and medium VL networks (Figure 4A). On the other hand, the network of the increased VL displayed various correlations, where, mainly, *Lactobacillus* displayed negative correlations with other genera such as *Megasphera*, *Prevotella_9*, *Bifidobacterium*, among others.

### 3.6. Lactobacillus and Community State Type Distribution

**Figure 5:**
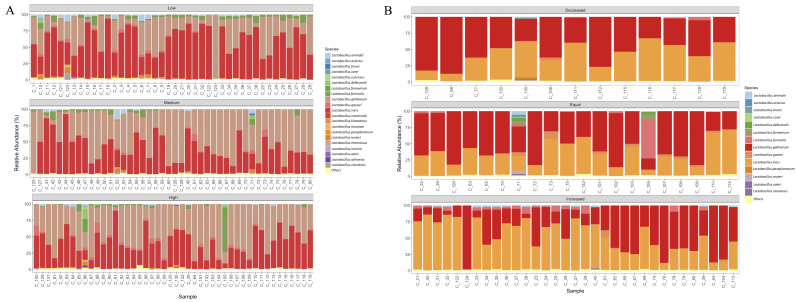
*Lactobacillus* composition of cervical samples. (**A**) Bar plots showing the main bacterial species by viral load, considering all follow-up time points (first and second), and categorizing women according to their VL despite their follow-up time point. (**B**) Bar plots showing the main bacterial species by viral load outcome. For this figure, we only considered the VL outcome at 12 months.

In the study population, *Lactobacillus* represented the most abundant genus (51.0%) of the cervical microbiota, although we observed some differences in their abundance according to the analyzed group (Figure 5). For the follow-up variable, we observed a higher relative abundance of *Lactobacillus* in the follow-up at 12 months compared to the baseline (*p* = 0.0029 (Mann–Whitney)) (Figure S8). A significant increase in *Lactobacillus* in women with high VL was found (*p* = 0.024 (Kruskal–Wallis)); specifically, the post hoc Dunn test exhibited significant differences between low and medium VL (*p* adjusted= 0.019, (Dunn test)) (Figure 5A). On the other hand, for the VL outcome, we observed no significant differences in the abundance of the *Lactobacillus* communities (*p* = 0.7065 (Kruskal–Wallis)) (Figure 5B).

Additionally, we evaluated the association between the *Lactobacillus* depletion (<60%) and the two variables (VL and VL outcome) using logistical regression analysis. The results show a reduction in the odds ratio for the *Lactobacillus* depletion in the medium VL (adjusted OR 0.12; 0.03–0.44 95%CI) and high VL (adjusted OR 0.23; 0.07–0.78 95%CI) (Table 1).

Regarding the *Lactobacillus* species, *L. gallinarum* was the most abundant species in women of the first follow-up (*p* = 0.040) and women with high VL (*p* = 0.028). The second most abundant species was *L. iners*, displaying a high abundance in women of the second follow-up (*p* = 0.1367) and with low VL (*p* = 0.0100) (Figure S9).

The different community state types (CST) were proposed to categorize the cervical microbiota according to the dominance of a particular *Lactobacillus* species (*L. crispatus*, *L. gasseri*, *L. iners*, and *L. jensenii*) [14,15,16]. We described the most common CST in the study population, where the results showed two principal CST. CST IV was the one with the higher occurrence (89.4%, 118/132), this type was characterized by a lack of *Lactobacillus* and a dominance of strict or facultative anaerobes (principally *Gardnerella*, *Prevotella*, *Atopobium*, *Sneathia*). On the contrary, CST III was the one with the minor occurrence, and was dominated by the species *L. iners* (10.6%, 14/132). Furthermore, we assessed the association between CST III and the two variables (VL and VL outcome), and the results exhibited a positive association between the CST dominated by *L. iners* and a low VL (adjusted OR 6.06: 1.06–4.55 95%CI) (Table 2).

## 4. Discussion

Herein, we indicate that women with hrHPV infections have a higher diversity of bacterial communities (Figure 2), which is consistent with transversal [13,17,32] and longitudinal studies [13,14] that reported an association between viral infection and a higher microbiota diversity. However, variations in diversity and abundance were observed according to viral load, being significantly higher in the presence of low copies of the virus (Figure 2 and Table S7).

The number of copies of hrHPV has been proposed as a biological indicator of CC; however, viral persistence in a host is required for a lesion to occur, so low VL could be a viral strategy to ensure such infections are silent and do not alert the immune system [33]. A low hrHPV VL has been associated with the integration of the viral genome, resulting in the expression of the hrHPV E6 protein [34,35]; this viral protein modulates the availability of tryptophan by decreasing its concentration in the extracellular medium. Studies have also shown an association between tryptophan depletion and CST IV cervical microbiota (characterized by the enrichment of anaerobic bacteria and states of dysbiosis), where a greater bacterial diversity and depletion in *Lactobacillus* is observed [36,37,38]. Moreover, the expression of the E6 oncoprotein favors deregulation in gene expression and the accumulation of mutations in the host genome that promote progression to cervical malignancy [39].

Our results show that women with low VL are characterized by a decrease in *Lactobacillus* (Table 1), and reports suggest that a decrease in *Lactobacillus* abundance is related to cervical lesions. Additionally, women with intraepithelial lesions (LSIL or HSIL) present low viral loads, which may be explained by the integration of the HPV genome. The genome integration is associated with a downregulation in viral DNA synthesis, which affects the immune system activation and diminishes the probability of eliminating the infection. Nevertheless, future studies are required to comprehend the role of individual bacterial species in the viral load dynamics.

Interestingly, our results show a remarkably low abundance of *L. crispatus* and the absence of *L. jensenii*, which have both been reported as beneficial *Lactobacillus* species to the host [40,41]. In contrast, we found a high abundance of *L. iners* and *L. gallinarum* (Figure 5). *Lactobacillus iners* does not produce D-lactic acid, essential in the acidification of the vaginal environment and protection against the production of particular metabolites (e.g., extracellular matrix metalloproteinase inducer and other matrix metalloproteinases). Thus, the lack of this species could favor the decomposition of the extracellular matrix, favoring the proliferation of pathogens in the cervical epithelium [42,43]. This *Lactobacillus* species has been described as a vaginal symbiont promoting a healthy vagina, and as an opportunistic pathogen associated with minor protection against vaginal dysbiosis. *Lactobacillus iners* can easily adapt to the fluctuating vaginal niche, and has been isolated from both healthy and bacterial vaginosis states [43,44]. Therefore, the latter could explain its high abundance in the study population and its association with hrHPV, mainly in the low VL group (Figure 3 and Table 2).

*Lactobacillus gallinarum* exhibited a high prevalence in the analyzed population; this species has been reported at the intestinal level, where it has antitumor activity by promoting the apoptosis of colorectal tumor cells [45]. At the vaginal level, it has also been found in the Chinese population, where it seems to be a protective factor against primary ovarian failure (POF) [46]. However, to date, its significant distribution has not been related to infection, or lack of, by hrHPV [47]. Differences in the predominant species of *Lactobacillus* have been described according to the evaluated population or ethnic group [48,49]. The high presence of *L. gallinarum* in this study could indicate the adaptation of *Lactobacillus* species to particular populations; this can be partly explained by genetic factors of the host (such as variations in the immune system), sexual behavior, and cultural characteristics [48,49,50]. Nevertheless, additional studies that include a heterogeneous population, quantification (e.g., with qPCR), and culture isolation coupled with genomic characterization are necessary to understand the vaginal composition of *Lactobacillus*.

Some bacterial genera presented a differential abundance according to the viral load (Figure 3). We found a higher abundance of *Prevotella* in hrHPV infections with a low VL; this genus has been related to vaginal microbiota homeostasis alteration by providing nutrients to other anaerobes such as *G. vaginalis* and *P. anaerobius* [51]. Alternatively, *Gardnerella* was found to have an increased abundance in women with high VL (Figure 3); in particular, the species *G. vaginalis* is associated with bacterial vaginosis (BV), and its presence is related to infertility, premature labor, and adverse neonatal outcomes, among others [46]. Alterations in the cervical microbiota derived from the presence of these bacteria lead to modifications in the local immune response, with an increase in IL-6, IL-8, and tumor necrosis factor (TNF)-α, which promote chronic inflammatory states [46].

Studies of the cervical microbiome have been directed primarily towards understanding the relationship between particular bacterial taxa and the presence of hrHPV. One of the main findings of this study is the significant variation in the composition and diversity of bacterial communities based on the hrHPV load; this could suggest the presence of selective pressures that contribute to the change in the microbiota at the level of the cervix. However, one of the limitations of this study is the absence of hrHPV-negative women (as a control group) and the use of only two follow-up points for the patients. Another limitation is the small sample size; in this study, we performed convenience sampling, with a proportional number of samples among groups. Despite these limitations, we found significant associations. On the other hand, from the 14 HPV types described as high-risk, we quantified six high-risk types that were previously reported as prevalent in the Colombian population. Future analyses could consider including a higher number of HPV types, allowing researchers to capture the HPV diversity and coinfection events. Finally, the V4 region of the 16S rRNA gene was sequenced in this study; although several studies have reported successful taxonomic assignation results when targeting the V4 region, short-read sequencing platforms may not achieve the complete taxonomic resolution provided by other sequencing methodologies, such as full-length 16S rRNA gene sequencing. These limitations should be considered for the design of future studies, in such a way that they provide a broader picture of the dynamics of microbial communities and the viral load.

## 5. Conclusions

The association between hrHPV and CC has been clearly established, and considerable progress has been made in our understanding of it. Regardless, it remains to be revealed why only a few women infected with the virus develop cervical lesions, and the influence of additional factors, such as bacterial communities, in this process. The analysis of the coexistence with other microorganisms (beneficial and pathogenic), and their role in the dynamics of hrHPV, will contribute to the development of new approaches and the planning of efficient strategies for neoplasia control. Finally, this study provides valuable information on differentially present members in the cervical microbiota of women with distinct hrHPV loads, which could become biomarkers in the natural history of the infection.

## Figures and Tables

**Table 1:** Results of the logistic regression to determine the variables associated with the *Lactobacillus* species depletion (<60%) according to the evaluated groups.

Variable	Univariate Analysis			Multivariate Analysis		
	OR	95%CI	*p*-Value	ORa ^1^	95%CI	*p*-Value
**Viral Load**						
Low	Reference			Reference		
Medium	**0.13**	**0.03–0.45**	**0.001**	**0.12**	**0.03–0.44**	**0.001**
High	**0.22**	**0.07–0.73**	**0.013**	**0.23**	**0.07–0.78**	**0.018**
**Viral Load Outcome**						
Decrease	Reference			Reference		
Equal	0.64	0.23–1.79	0.403	0.58	0.20–1.64	0.312
Increase	1.05	0.40–2.75	0.907	0.93	0.35–2.46	0.892

^1^ OR adjusted age and colposcopy status. CI, confidence interval. Significant *p*-values in bold.

**Table 2:** Results of the logistic regression to determine the variables associated with the community state type (CST) III according to the evaluated groups.

Variable	Univariate Analysis			Multivariate Analysis		
	OR	95%CI	*p*-Value	ORa ^1^	95%CI	*p*-Value
Second	Reference			Reference		
**Viral Load**						
Low	4.56	0.85–6.41	0.076	**6.06**	**1.06–4.55**	**0.042**
Medium	2.07	0.39–10.89	0.387	2.35	0.44–12.5	0.316
High	Reference			Reference		
**Viral Load Outcome**						
Decrease	0.85	0.17–4.15	0.843	0.88	0.18–4.35	0.881
Equal	0.90	0.21–3.82	0.897	0.96	0.22–4.12	0.967
Increase	Reference			Reference		

^1^ OR adjusted age and colposcopy status. CI, confidence interval. Significant *p*-values in bold.

## Data Availability

The original contributions presented in the study are included in the article/Supplementary Material; further inquiries can be directed to the corresponding author. The raw data of this study have been deposited in the European Nucleotide Archive (ENA) at EMBL-EBI project accession PRJEB55377.

## Supplementary Materials

The following are available online at https://www.mdpi.com/article/10.3390/v14122674/s1. Figure S1: Flowchart of study design. Figure S2: Median of viral load in groups of study regarding follow-up. The dotted line indicates the median; the box represents the interquartile range (IQR). The whiskers extending from the boxes are the upper and lower limits. Circle markers represent extreme values. Figure S3: Distribution of viral load in groups of study. Left panel, median of viral load regarding follow-up; right panel, schematic representation of women according to their viral load at the beginning of the study and variation in viral load for the second follow-up. Figure S4: Rarefaction curve showing the species richness in function of depth read by sample. Figure S5: Microbial composition of cervical samples. Bar plots showing the main archaea genera by follow-up. (A) Bar plots showing the archaea genera by viral load, considering all the follow-up time points (first and second), and categorizing women according to their VL independent of their follow-up point. (B) Bar plots showing the archaea genera by viral load outcome. For this figure, the viral load outcome at 12 months is considered. Figure S6: Microbial composition of cervical samples. Bar plots showing the main bacterial genera by follow-up. The samples correspond to the same individual, and the first follow-up is shown at the top panel, and the second at the bottom panel. Additionally, samples are ordered according to the VL at the beginning of the study; first, those with low VL (samples C_1 to C_20, C_121, and C_122), then those with medium VL (samples C_41 to C_60, C_125, and C_127), and finally, those with high VL (samples C_81 to C_100, C_129, and C_131). (B) Distribution of each bacterial genera by follow-up. Figure S7: Comparison between groups of relative abundance of the 20 main bacterial genera with their phyla. (A) Boxplot showing the differences between viral load by relative abundance of each genus. (B) Boxplot showing the differences between viral load outcome by relative abundance of each genus. Figure S8: *Lactobacillus* species composition of cervical samples by follow-up. The samples corresponding to the same individual and the first follow-up are shown in the top panel, and, in the bottom panel, the second follow-up. Additionally, data according to the VL at the beginning of the study are shown; first, those with low VL (samples C_1 to C_20, C_121, and C_122), then, those with medium VL (samples C_41 to C_60, C_125, and C_127) and finally, those with high VL (samples C_81 to C_100, C_129, and C_131). Figure S9: Heatmap of *Lactobacillus* species frequency regarding follow-up, viral load, and viral load outcome. Table S1: The study population’s sociodemographic and clinical characteristics and risk factors upon enrolment in the study. Table S2: Number of reads of the assigned bacterial taxonomic ranks per sample (DataSheet). Table S3: Number of reads and relative abundance (%) of the identified bacterial phyla (DataSheet). Table S4: Number of reads and relative abundance (%) of the identified bacterial genera (DataSheet). Table S5: Number of reads of the different archaea taxonomic ranks per sample (DataSheet). Table S6: Number of reads and relative abundance (%) of the identified archaea genera (DataSheet). Table S7: Multilevel mixed-effects linear regression to estimate the effect of the variables on the diversity.
