# Supplementary material for: Changes in the Cervical Microbiota of Women with Different High-Risk Human Papillomavirus Loads
Source: Viruses. 2022 Nov 29;14(12):2674. doi: 10.3390/v14122674 (PMC9781391; doi:10.3390/v14122674)
Supplement: Supplementary file 1 [file viruses-14-02674-s001.zip › viruses-1986669-Supplementary Material.pdf]

Supplementary Material

Table S1. The study population's sociodemographic and clinical characteristics and risk factors on enrolment in the study

		Low Viral Load		Medium Viral Load		High Viral Load		p-value
**CHARACTERISTICS**		**Median**	**IQR**	**Median**	**IQR**	**Median**	**IQR**	
**Age in years**		45	23	38.5	15	47	8	
**Age at first intercourse in years**		16.5	3	17	5	18	4	
	**Categories**	***n***	***%***	***n***	***%***	***n***	***%***	
**Average monthly income[1]**	≤ minimum	20	90.9	20	90.9	16	76.2	0.364
	> minimum	2	9.1	2	9.1	5	23.8	
**Marital status[2]**	Status 1	2	9.1	2	9.1	1	4.5	1.000
	Status 2	20	90.9	20	90.9	21	95.5	
**Number of sexual partners**	1	6	27.3	10	47.6	9	45.0	0.301
	2-3	13	59.1	6	20.6	9	45.0	
	> 3	3	13.6	5	23.8	2	10.0	
**Pregnancies**	0-1	1	4.5	1	4.5	2	9.1	1.000
	≥ 2	21	95.5	21	95.5	20	90.9	
**Contraceptive method3**	No method	3	14.3	11	53.4	8	38.1	0.046
	Hormonal	4	19.0	4	19.0	1	4.8	
	Others	14	66.7	6	28.6	12	57.1	
**Abortions**	No	18	55.6	6	46.2	10	62.5	0.678
	Yes	8	44.4	7	53.8	6	37.5	
**Colposcopy result**	Negative	15	68.2	19	86.4	19	86.4	0.047
	LSIL	6	27.3	3	13.6	3	13.6	
	HSIL	1	4.5	0		0		

[1] Colombian minimum average monthly income is around US$ 280.
[2] The marital status 1: category included single, divorced women and widows and marital status 2: included married women and women living with a partner/free union.
[3] The contraceptive method 'others' included barrier methods and surgery.

Table S2. Number of reads of the assigned bacterial taxonomic ranks per sample (DataSheet).

Table S3. Number of reads and relative abundance (%) of the identified bacterial phyla (DataSheet).

Table S4. Number of reads and relative abundance (%) of the identified bacterial genera (DataSheet).

Table S5. Number of reads of the different archaea taxonomic ranks per sample (DataSheet).

Table S6. Number of reads and relative abundance (%) of the identified archaea genera (DataSheet).

Table S7. Multilevel mixed-effects linear regression to estimate the effect of the variables on the diversity.

	Shannon index			Simpson index		
**Variable**	**Coeff.**	**95%CI**	**p-value**	**Coeff.**	**95%CI**	**p-value**
**Follow-up**						
First	Reference			Reference		
Second	-38.31	-94.92-28.30	0.260	**-0.41**	**-0.56-(-)0.25**	**0.000**
**Viral Load**						
Low	Reference			Reference		
Medium	-30.18	-80.24-20.57	0.244	**-0.34**	**-0.46-(-)0.22**	**0.000**
High	-39.31	-90.10-11.41	0.129	**-0.17**	**-0.29-(-)0.05**	**0.004**
**Follow-up/Viral Load**						
First/ Low	58.52	21.42-95.61	0.202	**0.89**	**0.80-0.97**	**0.000**
Second/ Low	20.20	-31.78-78.20	0.492	**0.47**	**0.34-0.61**	**0.000**
First/Medium	28.33	-8.76- 65.42	0.133	**0.54**	**0.46-0.63**	**0.000**
Second/ Medium	29.34	-11.66- 70.35	0.159	**0.58**	**0.49-0.68**	**0.000**
First/ High	19.17	-17.92- 56.26	0.308	**0.71**	**0.63-0.80**	**0.000**
Second/High	17.36	-10.49- 45.22	0.220	**0.62**	**0.56-0.69**	**0.000**

Coeff= Coefficient

Supplementary Figures

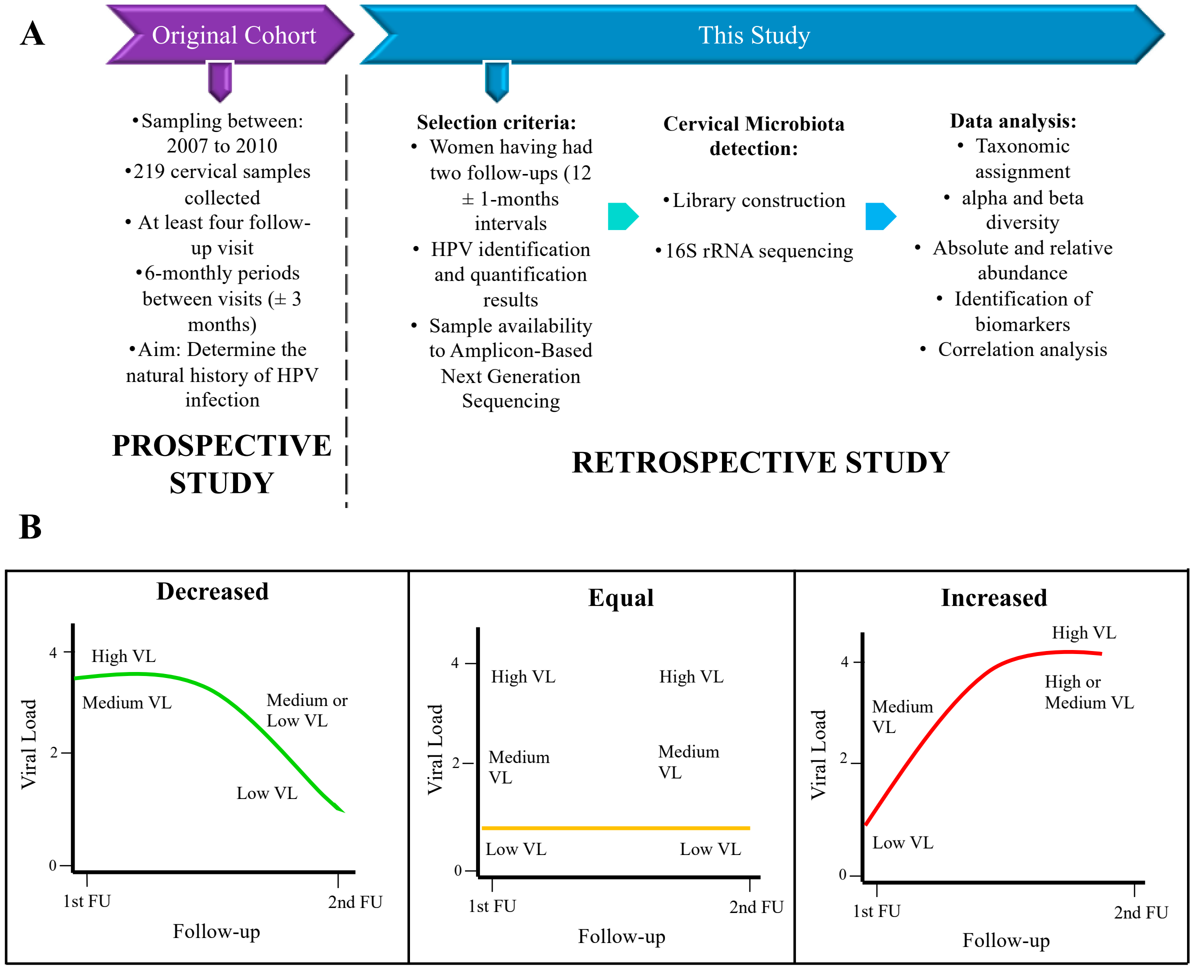

Figure S1. Flowchart of study design and definition of viral load outcome.

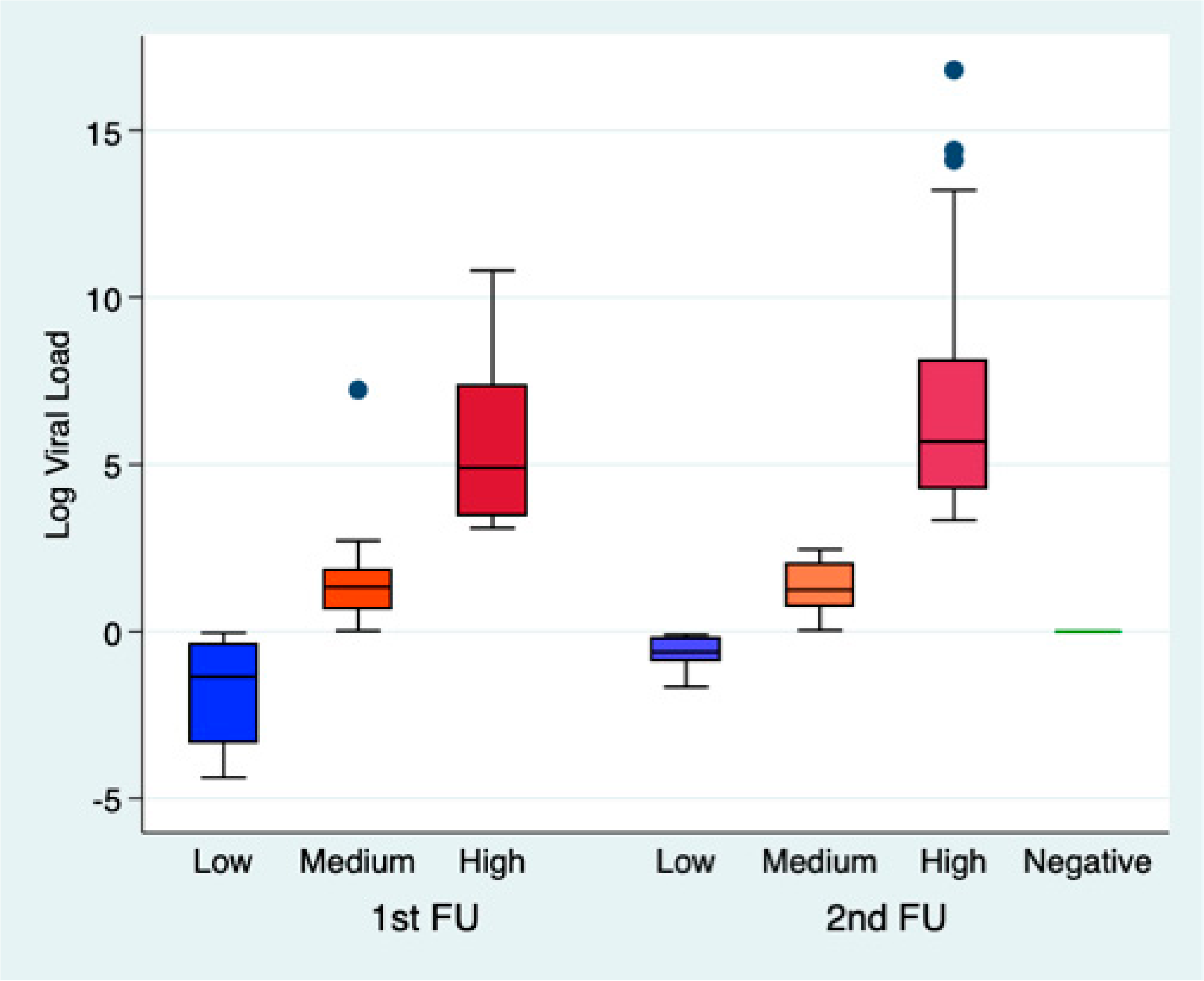

Figure S2. Median of viral load in groups of study regarding follow-up. The dotted line indicates the median; the box represents the interquartile range (IQR). The whiskers extending from the boxes are the upper and lower limits. Circle markers represent extreme values.

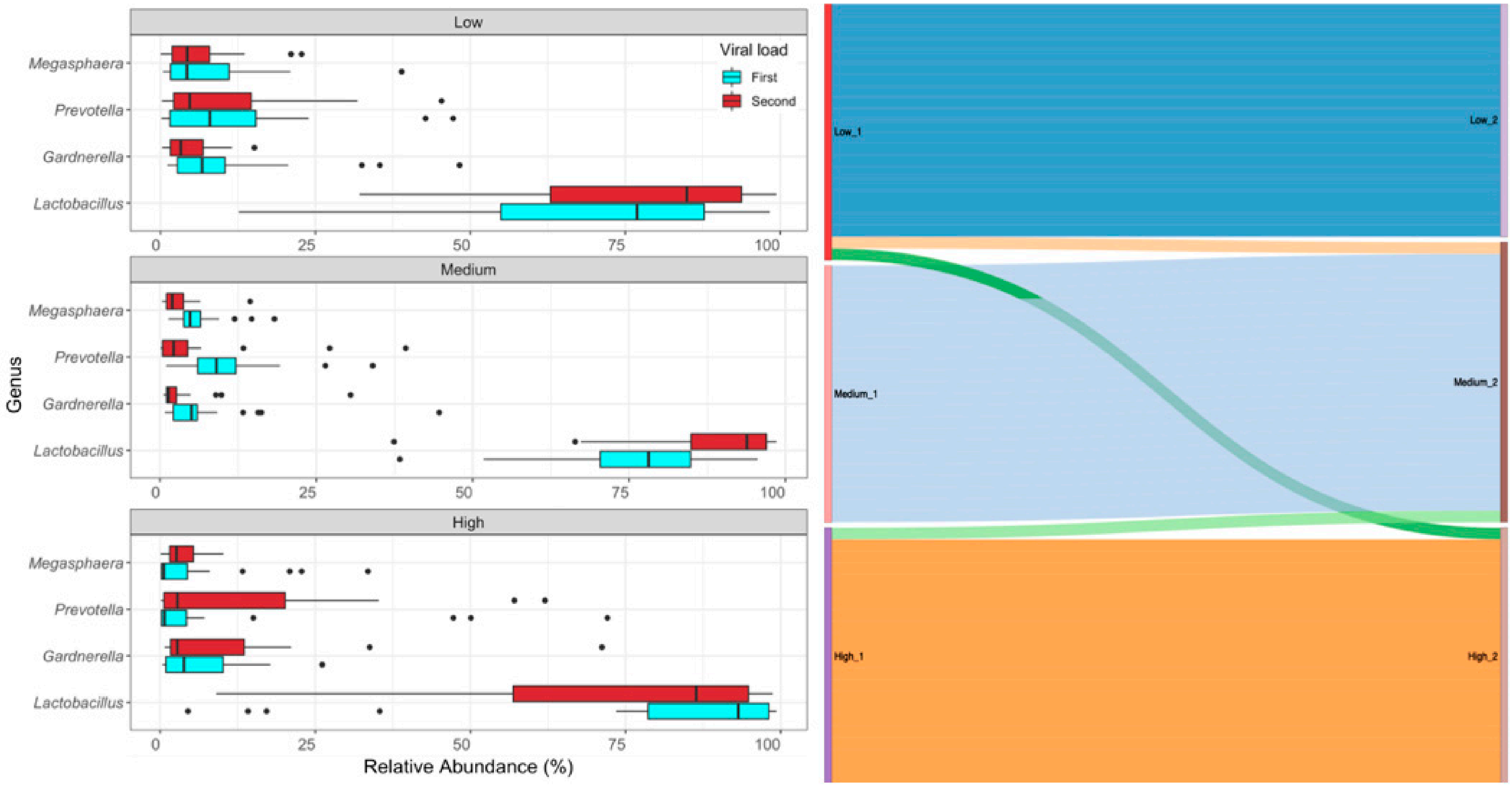

Figure S3. Distribution of viral load in groups of study. Panel left: median of viral load regarding follow-up. Panel right: schematic representation of women according to their viral load at the beginning of the study and variation of viral load for the second follow-up.

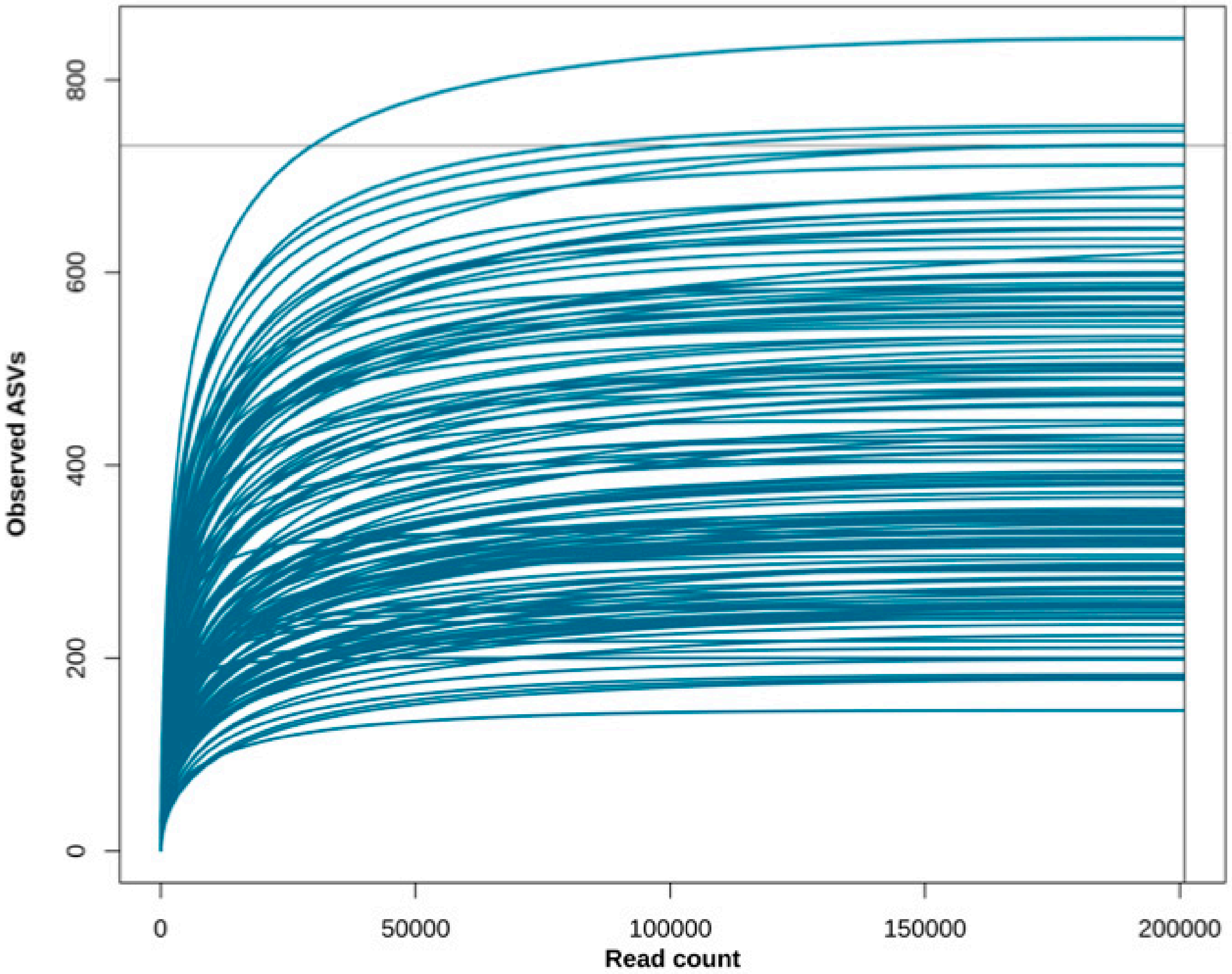

Figure S4. Rarefaction curve showing the species richness in function of depth read by sample.

A

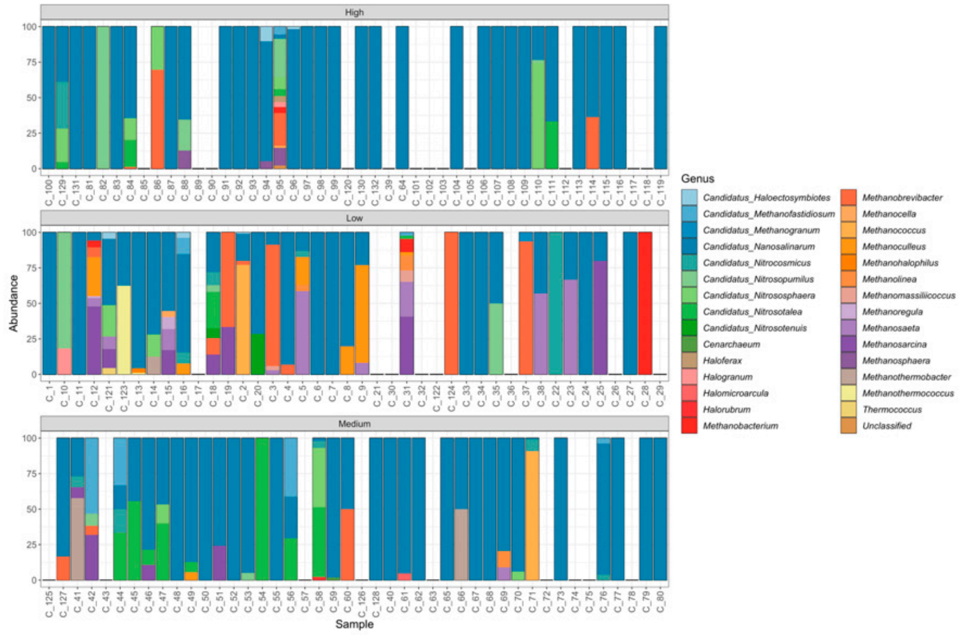

B

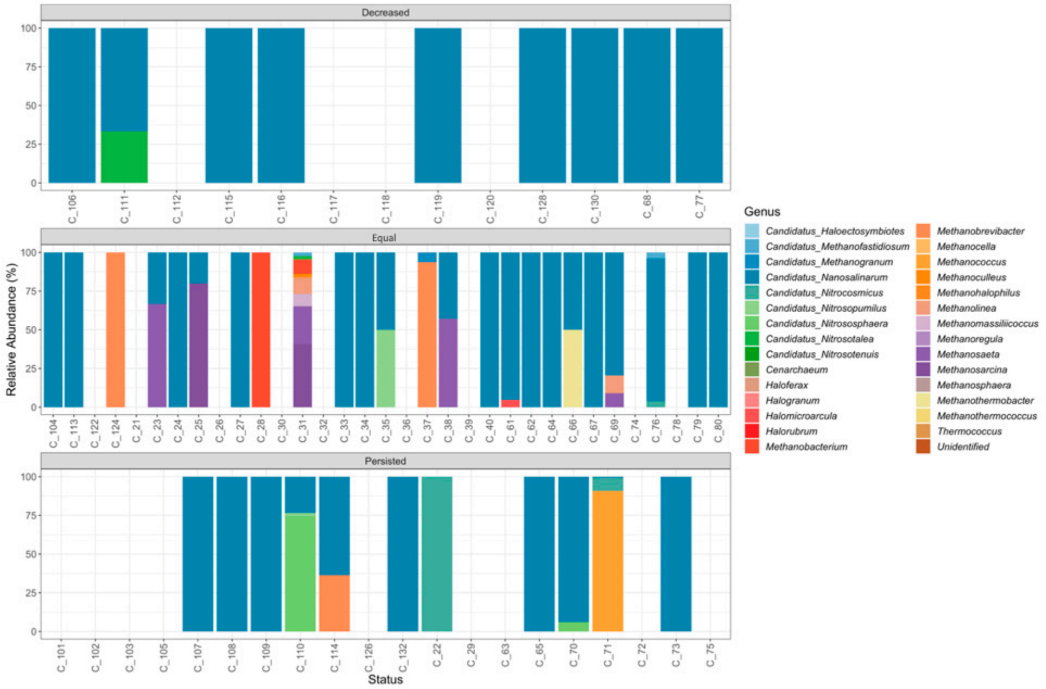

Figure S5. Microbial composition of cervical samples. bar plots showing the main archaea genera by follow-up. (A) Bar plots showing the archaea genera by viral load, considering all the follow-up time points (first and second), and categorizing women according to their VL independent of their follow-up point (B) Bar plots showing the archaea genera by viral load outcome. For this figure the viral load outcome at 12-months is considered.

A

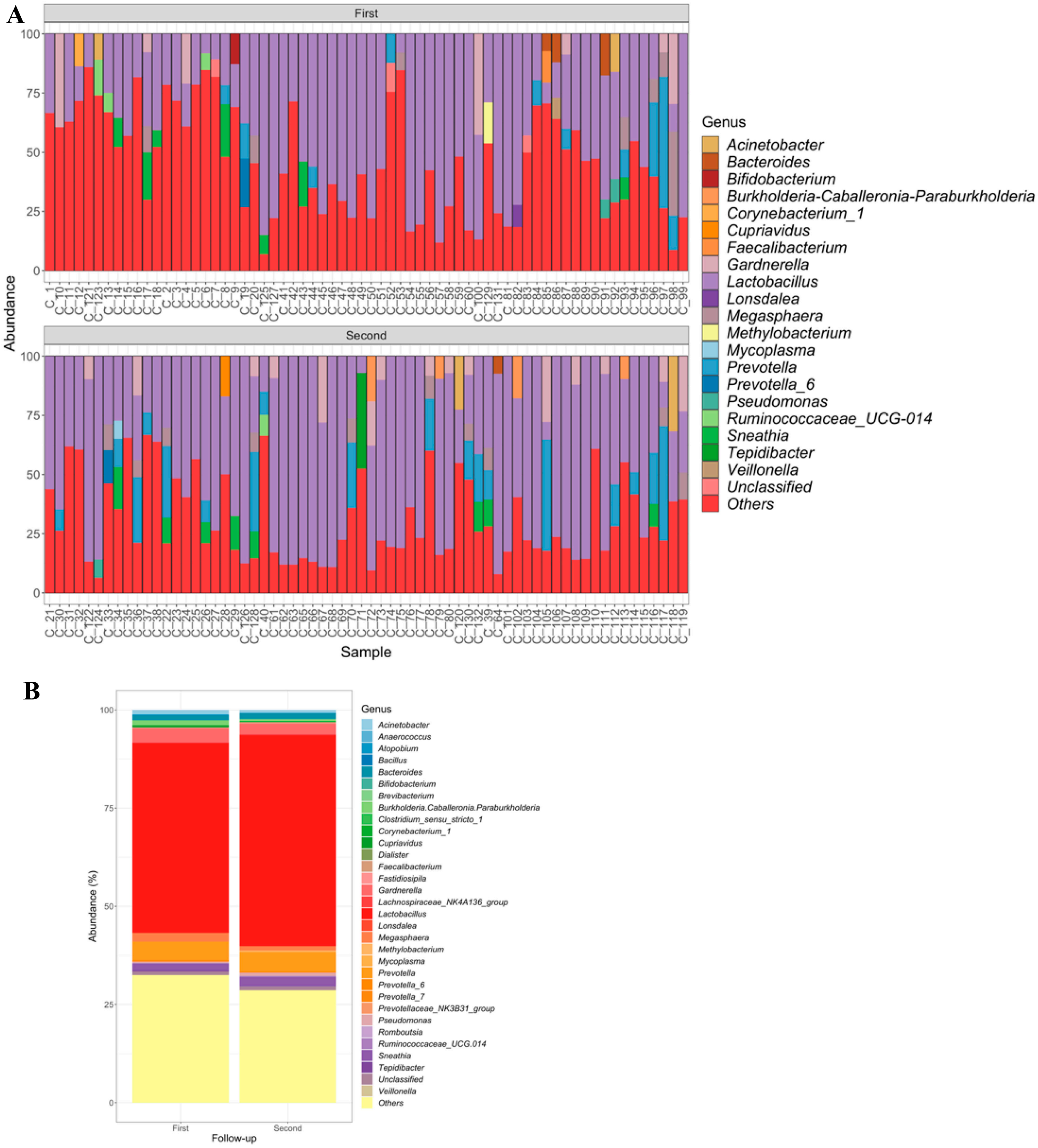

Figure S6. Microbial composition of cervical samples. Bar plots showing the main bacterial genera by fol-low-up. The samples correspond to the same individual, and the first follow-up is shown at the top panel, and the second, at the bottom panel. Additionally, samples were ordered according to the VL at the beginning of the study: first those with low VL (samples C_1 to C_20, C_121 and C_122), then those with medium VL (samples C_41 to C_60, C_125-and C _127), and finally those with high VL (samples C_81 to C_100, C_129 and C_131). (B) Distribution of each bacterial genera by follow-up.

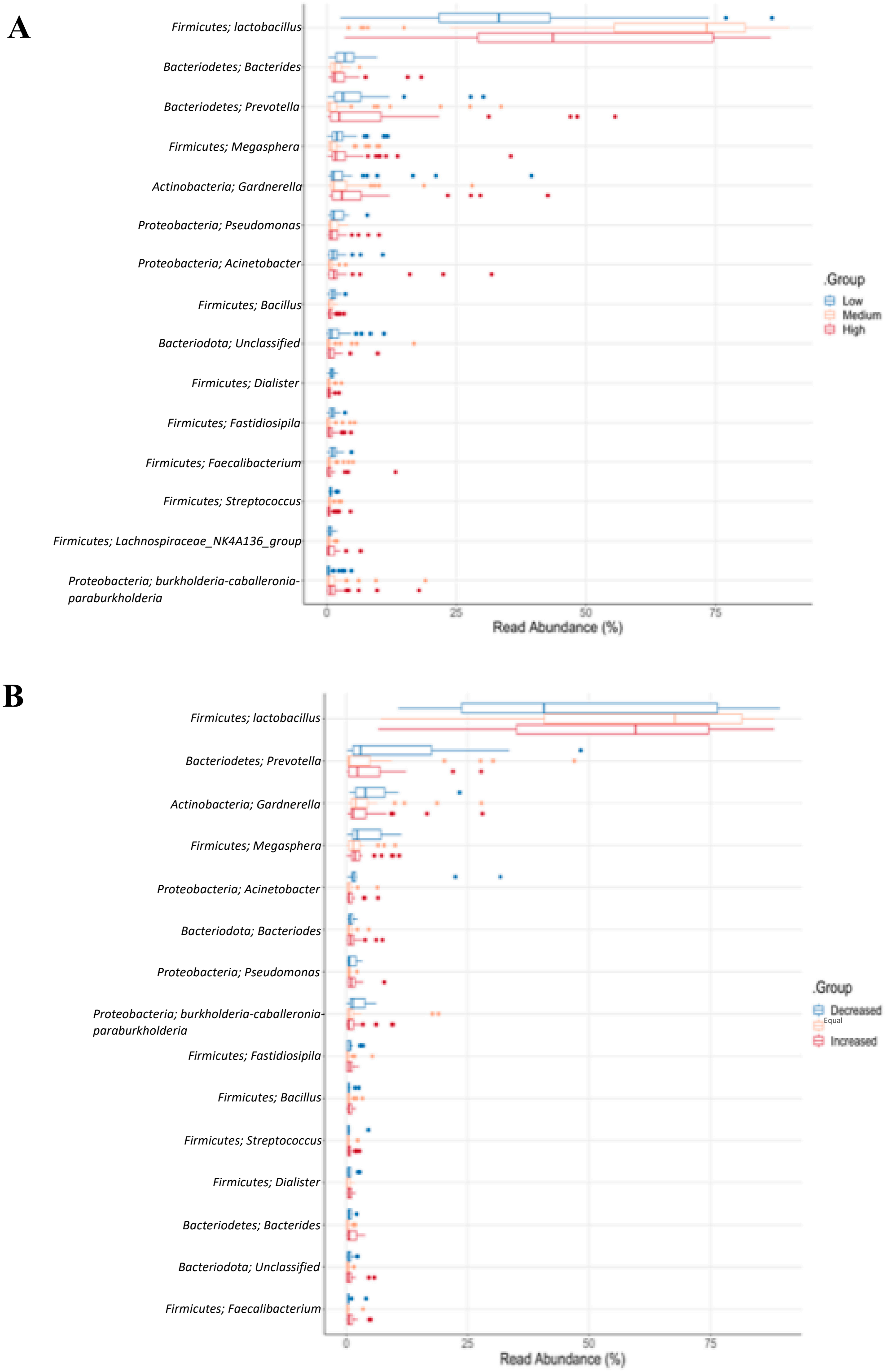

Figure S7. Comparison between groups of relative abundance of the 20 main bacterial genera with their phyla. (A) Boxplot showing the differences between viral load by relative abundance of each genus. (B) Boxplot showing the differences between viral load outcome by relative abundance of each genus.

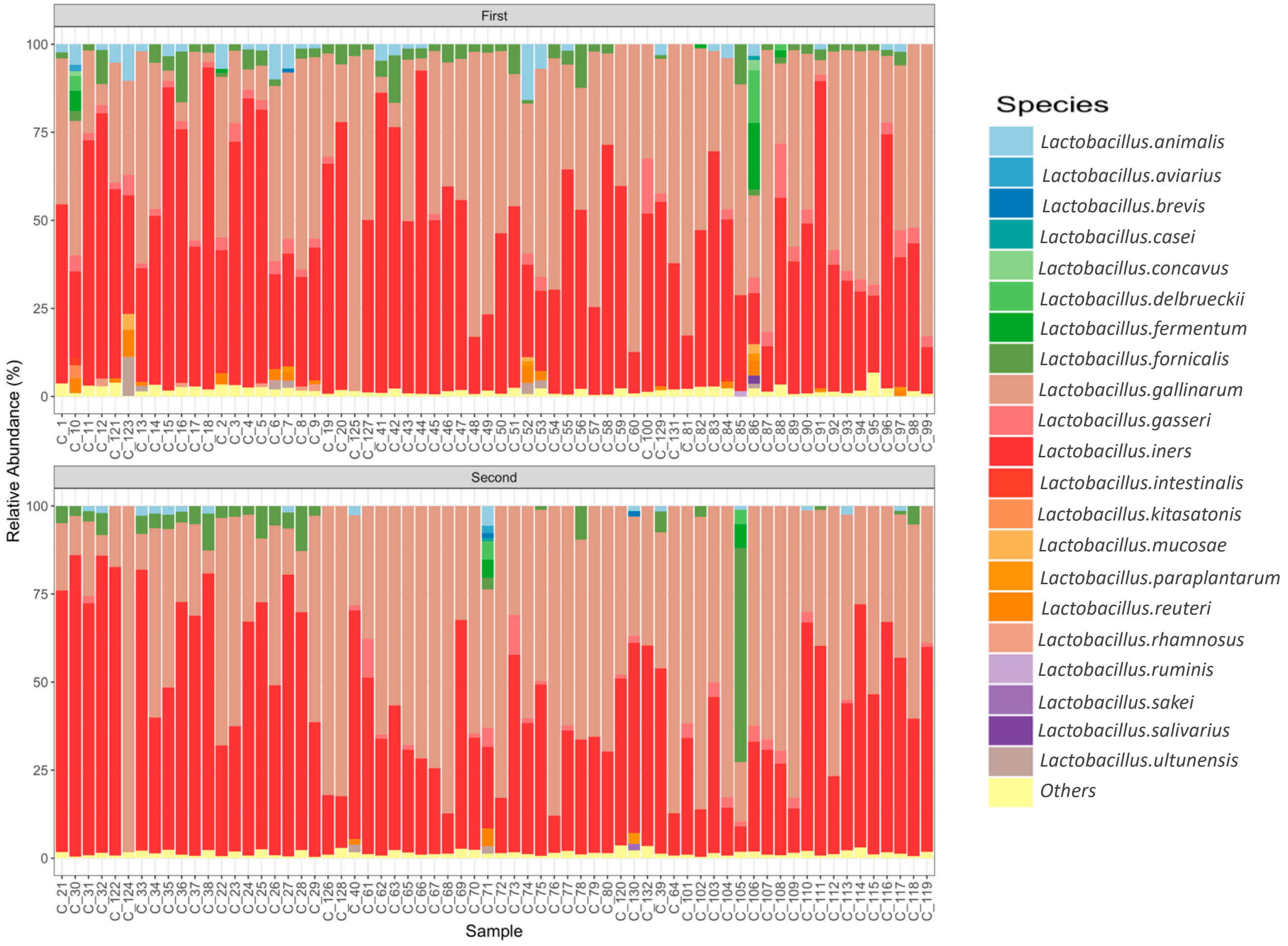

Figure S8. *Lactobacillus* species composition of cervical samples by follow-up. The samples correspond the same individual and the first follow-up is shown at the top panel and, at the bottom panel, the second. Additionally, according to the VL at the beginning of the study: first those with low VL (samples C_1 to C_20, C_121 and C_122), then those with medium VL (samples C_41 to C_60, C_125-and C _127)), and finally those with high VL (samples C_81 to C_100, C_129 and C_131).

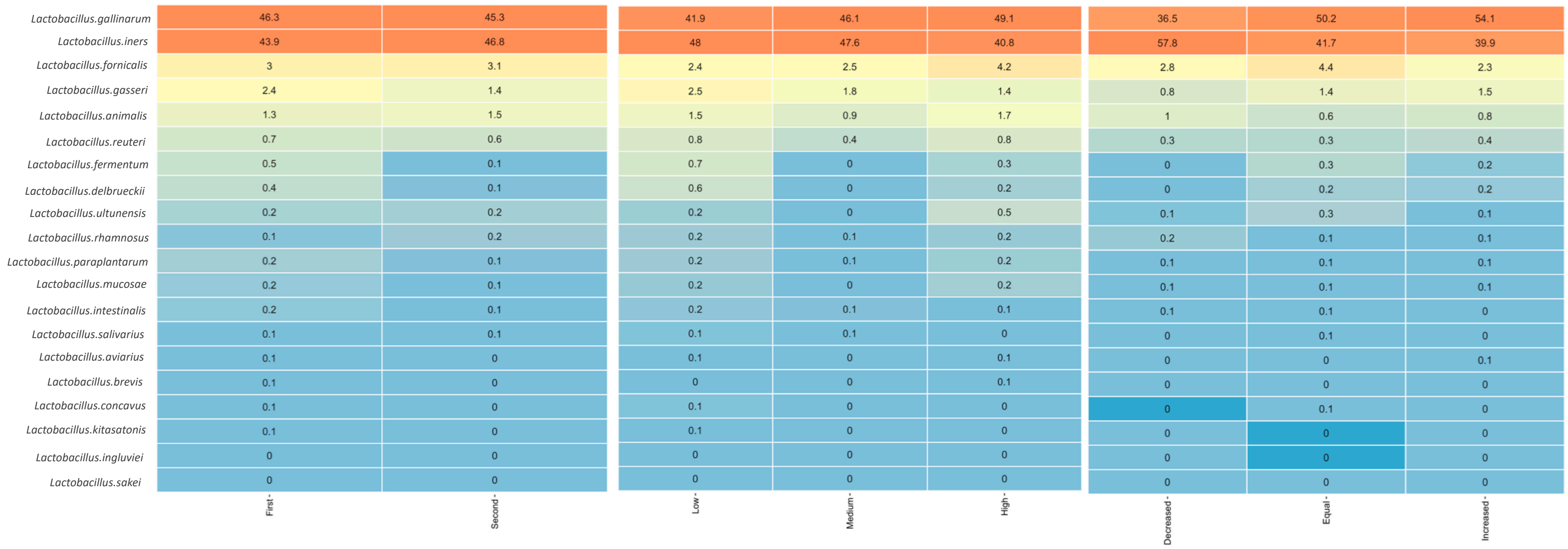

Figure S9. Heatmap of *Lactobacillus* species frequency regarding follow-up, viral load, and viral load outcome.
